# Tinnitus among post-9/11 veterans: psychiatric comorbidity and associations with health and functional outcomes

**DOI:** 10.1080/20008066.2026.2623718

**Published:** 2026-02-26

**Authors:** Beth M. McCormick, Sara E. Wallander, Mark W. Miller, Xiang Zhao, William P. Milberg, Catherine B. Fortier, Erika J. Wolf

**Affiliations:** a National Center for PTSD at VA Boston Healthcare System, Boston, MA, USA; b Department of Psychiatry, Boston University Chobanian & Avedisian School of Medicine, Boston, MA, USA; c Department of Biostatistics, Boston University School of Public Health, Boston, MA, USA; d Translational Research Center for TBI and Stress Disorders and Geriatric Research Educational and Clinical Center, VA Boston Healthcare System, Boston, MA, USA; e Department of Psychiatry, Harvard Medical School, Boston, MA, USA

**Keywords:** Tinnitus, PTSD, traumatic brain injury, veteran, chronic pain, functioning, Tinnitus, TEPT, traumatismo craneoencefálico, veterano, dolor crónico, funcionamiento

## Abstract

**Background:** Tinnitus is common among Veterans and is associated with PTSD and traumatic brain injury (TBI).

**Objective:** To characterize tinnitus and examine the overlap of tinnitus, PTSD, and TBI and their relative associations with sleep disturbance, chronic pain, and cognitive and functional impairment.

**Method:** Participants were 735 primarily young (*M* = 34.7, *SD* = 9.1), male (90.2%) post-9/11 US Veterans. Hierarchical regressions were performed to determine whether additional variance in cross-sectional and longitudinal health outcomes was explained by tinnitus beyond PTSD and TBI.

**Results:** Tinnitus was common (67.5%) and evidenced substantial comorbidity with both PTSD and TBI in 35.6% of the sample. Tinnitus explained an additional 1.9-3% of the variance (both Δ *p* < .001) in pain (β = .184, *p* < .001) and functional impairment (β = .145, *p* < .001) after accounting for PTSD severity and number of TBIs and adjusting for multiple testing. PTSD significantly predicted all outcomes (βs = −.226 -.657, all *p*s < .001). Tinnitus showed stability over time but did not predict later pain or functional impairment.

**Conclusions:** Results suggest tinnitus is common among young treatment-seeking Veterans and highlight the need for future research aimed at reducing the toll of this chronic condition. Tinnitus should be evaluated alongside PTSD and TBI, as it may be associated with more negative health outcomes, even among those already at increased risk due to PTSD and TBI. Clinicians should be informed about best practices for managing tinnitus in efforts to improve health and well-being.

## HIGHLIGHTS

- Tinnitus was endorsed by over two-thirds of this cohort of post-9/11 Veterans; it was comorbid with both PTSD and TBI in over one-third.
- Tinnitus was associated with incrementally worse self-reported pain and functional impairment, even after accounting for the effects of concurrent PTSD and TBI.
- Tinnitus was not related to future worsening of pain and functional impairment beyond baseline levels.

## Abstract (es)

Antecedentes: El tinnitus es común entre los veteranos y se ha asociado con el trastorno de estrés post traumático (TEPT) y el traumatismo craneoencefálico (TEC). Objetivo: Este estudio caracterizó el tinnitus y examinó su superposición con TEPT y TEC, así como sus asociaciones relativas con consecuencias de salud en las áreas de alteración del sueño, dolor crónico y deterioro cognitivo y funcional. Método: Participaron 735 veteranos estadounidenses, principalmente jóvenes (*M* = 34,7; *DE* = 9,1), varones (90,2%), tras el 11 de septiembre (9/11), en un estudio longitudinal en curso. Se realizaron regresiones jerárquicas para determinar si el tinnitus explicaba alguna varianza adicional en los resultados de salud transversales y longitudinales, más allá del TEPT y el TEC. Resultados: El tinnitus fue muy frecuente (67,5%) y presentó una comorbilidad significativa con TEPT y TEC en el 35,6% de la muestra. El tinnitus explicó entre un 1,9% y un 3% adicional de la varianza (ambos Δ *p* < .001) en el dolor (β = 0,184, *p* < .001) y el deterioro funcional (β = 0,145, *p* < .001) tras considerar la gravedad del TEPT y el número de TECs en las etapas iniciales del modelo y ajustar por pruebas múltiples. El TEPT predijo significativamente todos los resultados (βs = −0,226 - 0,657, todos los *p*s < 0,001). En los análisis de seguimiento longitudinal, el tinnitus mostró estabilidad a lo largo del tiempo, pero no predijo dolor ni deterioro funcional posteriores más allá del TEPT, el TEC y los niveles basales de estos resultados. Conclusiones: Los resultados sugieren que el tinnitus es común incluso entre veteranos jóvenes que buscan tratamiento y resaltan la necesidad de futuras investigaciones para reducir las consecuencias de esta afección crónica. El tinnitus debe evaluarse junto con el TEPT y el TEC, ya que puede estar asociado con más consecuencias negativas para la salud, incluso en personas con mayor riesgo debido al TEPT y el TEC. Los profesionales de la salud mental deben estar informados sobre las mejores prácticas para el manejo del tinnitus con el fin de mejorar la salud y el bienestar.

## 1. Introduction

Tinnitus is defined as the ‘perception of sound that does not have an external source’ (National Institute on Deafness and Other Communication Disorders [NIDCD], 2023) and is most commonly described as ‘ringing in the ears,’ but may also manifest as ‘buzzing, hissing, whistling, swooshing’ or ‘clicking’ (American Tinnitus Association, n.d.). Reports of tinnitus are inherently subjective. Estimates of the prevalence of tinnitus generally range from 10-25% of US adults (Ausland et al., 2021; Bauer, 2018; Bhatt et al., 2016; de Gruy et al., 2024; Kim et al., 2015; NIDCD, 2023; Stegeman et al., 2021) and up to 67% in Veteran populations (Henry et al., 2019). Some estimates suggest that Veterans may be more than 2 times as likely to have tinnitus as non-Veterans (Folmer et al., 2011), likely reflecting their increased exposure to blasts and loud noise (e.g. gunfire, aircraft engines, exploding ordinances), which is part of the etiology of tinnitus (Bhatt et al., 2016; Henry et al., 2021; Shea et al., 2025; Theodoroff et al., 2015; Theodoroff & Konrad-Martin, 2020). Tinnitus was the most common service-connected disability among both new and all compensation VA recipients in 2024, with a total of 3,255,323 Veterans receiving service-connected compensation for tinnitus (US Department of Veterans Affairs, 2024). Thus, tinnitus carries a significant personal, societal, and financial burden.

Tinnitus is associated with poor physical and mental health outcomes, including sleep disturbances (Gu et al., 2022; Henry et al., 2019; Kim et al., 2015; Weingarten et al., 2024), chronic pain (Ausland et al., 2021; Coco et al., 2024a; Park et al., 2022; Swan et al., 2017), worse cognitive performance (e.g. executive function, memory; Clarke et al., 2020; Kuchinsky et al., 2020; Rossiter et al., 2006), and lower health-related quality of life/function (Henry et al., 2019; Park et al., 2022). Veterans with tinnitus are more likely than those without to suffer from mental health conditions such as depression, anxiety, and posttraumatic stress disorder (PTSD; Carlson et al., 2019; Coco et al., 2024b; Fagelson, 2007; Henry et al., 2019; Prewitt et al., 2021; Stegeman et al., 2021; Swan et al., 2017), though the temporal relationship between these conditions and tinnitus remains unclear.

Among Veterans with tinnitus, approximately one-third have comorbid PTSD (Coco et al., 2024a; Coco et al., 2024b; Fagelson, 2007). Among Veterans with PTSD, approximately 55% have comorbid tinnitus (Terhaag et al., 2021), while approximately 34-59% of Veterans with a history of traumatic brain injury (TBI) have comorbid tinnitus (Karch et al., 2016; Knoll et al., 2020; Le et al., 2024; MacGregor et al., 2020). In some cases, the overlap between PTSD, TBI, and tinnitus may reflect a shared etiology, such as a blast injury (Moring et al., 2018), and this may contribute to comorbidity among these conditions and overlap in their health correlates. The relative contribution of tinnitus as compared to PTSD and TBI in adverse health outcomes is unclear.

The aims of this study were two-fold. Our first objective was to examine patterns of comorbidity between tinnitus and PTSD, TBI, and related psychiatric conditions. Our second objective was to test if self-reported tinnitus contributed additional variance to the prediction of various health outcomes and cognitive performance metrics beyond that attributable to PTSD and TBI. We hypothesized that there would be substantial comorbidity across tinnitus, PTSD, and TBI and that tinnitus would be incrementally associated with (i.e. explain additional variance) neuropsychological performance (in the domains of working memory, explicit memory, and executive attentional function), sleep disturbance, pain severity, and functional impairment beyond associations with PTSD and TBI.

## 2. Methods

### 2.1. Participants and procedures

Data were from post-9/11 Veterans who took part in an ongoing longitudinal study, the Translational Research Center for TBI and Stress Disorders (TRACTS; McGlinchey et al., 2017), at sites in Boston, MA or Houston, TX. Participants underwent a comprehensive assessment that included structured psychiatric diagnostic interviews, a comprehensive neuropsychological battery, and self-report questionnaires. Exclusion criteria included psychotic and bipolar disorders, other acute psychiatric symptoms that would impede participation, neurological or cognitive disorder diagnoses (other than TBI or its acute effects), and active suicidal or homicidal ideation.

These analyses are based on a subset of *n* = 735 Veterans (out of a total *N* = 822 to date) with available data pertaining to PTSD diagnosis, TBI, and self-reported tinnitus from the baseline visit. Of those 735 Veterans, approximately 62% (*n* = 452) returned for a second assessment (T2) an average of 1.9 years later. The demographic characteristics of the cohort are listed in Table 1. Participants were predominately young (early 30s), and self-identified as white (71.7%), non-Hispanic (80.7%), and male (90.2%). The study was approved by the VA Boston Healthcare System IRB under protocol #1577628, and written informed consent was obtained for all participants prior to their involvement in the study. Qualified investigators may write to the corresponding author for instructions on how to apply to access data from the TRACTS data repository.

**Table 1.** Baseline sample demographic and clinical characteristics by tinnitus status (*n* = 735).

Variable	Tinnitus (*n* = 496)		No tinnitus (*n* = 239)		All	
	*M (SD)*	*n (%)*	*M (SD)*	*n (%)*	*M (SD)*	*n (%)*
Age	35.4 (9.1)**		33.4 (9.0)		34.7 (9.1)	
Sex (male)		449 (90.5)		214 (89.5)		663 (90.2)
Race						
Hispanic		81 (16.3)		50 (20.9)		131 (17.8)
White		371 (74.8)**		156 (65.3)		527 (71.7)
Black		63 (12.7)*		46 (19.2)		109 (14.8)
Asian		13 (2.6)		7 (2.9)		20 (2.7)
American Indian or Alaska Native		16 (3.2)**		0 (0)		16 (2.2)
Other		15 (3.0)		7 (2.9)		22 (3.0)
Unknown		39 (7.9)*		31 (13.0)		70 (9.5)
Education (years)	14.2 (2.1)		14.3 (2.2)		14.2 (2.1)	
Service branch						
Army		341 (68.8)		168 (70.3)		509 (69.3)
Navy		26 (5.2)		17 (7.1)		43 (5.9)
Air force		27 (5.4)*		24 (10.0)		51 (6.9)
Marines		118 (23.8)*		39 (16.3)		157 (21.4)
Neuro/psychiatric conditions						
Tinnitus						496 (67.5)
Current PTSD Dx		308 (62.1)***		103 (43.1)		411 (55.9)
Current PTSD severity	55.0 (28.2)***		38.0 (26.8)		49.4 (28.9)	
Hx of TBI (any)		395 (79.6)***		149 (62.3)		544 (74.0)
Hx of TBI (moderate/ severe)		28 (5.7)*		4 (1.7)		32 (4.4)
Hx of military blast		434 (87.5)***		169 (70.7)		603 (82.0)
n of lifetime TBIs	2.0 (2.5)***		1.3 (1.9)		1.8 (2.3)	
Lifetime TBI burden	3.3 (3.8)***		1.9 (2.6)		2.8 (3.5)	
Current mood Dx		172 (34.7)***		51 (21.3)		223 (30.3)
Current AUD Dx		75 (15.1)		33 (13.8)		108 (14.7)
Functional and health outcomes						
Current pain intensity	11.8 (9.4)***		5.6 (6.3)		9.5 (8.9)	
Current sleep disturbance	11.5 (4.7)***		9.0 (4.7)		10.7 (4.8)	
Current Fx impairment	27.2 (19.0)***		14.2 (14.9)		22.8 (18.7)	

Note. Neuropsychological factor scores for working memory, explicit memory, and executive attention are not included in this table because they are factor scores and therefore have a mean of 0. PTSD = posttraumatic stress disorder; Dx = diagnosis; Hx = history; Fx = functional; TBI = traumatic brain injury; AUD = alcohol use disorder; *p < .05; **p < .01, ***p < .001.

### 2.2. Measures

As part of a background health questionnaire created for the TRACTS study, tinnitus was assessed via a single self-report dichotomous item: ‘Do you have tinnitus (ringing in your ears)?’ This assessment approach is similar to other studies as systematic reviews suggest that single-item self-report measures are used frequently in tinnitus research (59-71%; McCormack, et al., 2016; Le et al., 2024).^1^

***Boston assessment of TBI (BAT-L).*** The BAT-L is a semi-structured clinical interview that assesses the total number and severity of TBIs experienced across the lifetime, including premilitary, blast-related, other military, and postmilitary TBI events (Fortier et al., 2014). BAT-L interviews were performed by doctoral-level psychologists and masters-level clinicians. In the original validation studies among 131 post-9/11 Veterans (a subset of Veterans in this cohort), the BAT-L evidenced strong interrater reliability (κs > 0.80) and good correspondance with the Ohio State University TBI Identification Method (Cohen κ = 0.89; Kendall τ-b = 0.95; Fortier et al., 2014). A total lifetime burden score that reflects both severity and occurrence can be calcuated. For the purposes of these analyses, to be able to compare results with prior studies, we utilized a sum score for the number of TBI events across all 4 categories (i.e. premilitary, blast-related, other military, postmilitary) to reflect the total number of TBIs experienced across the lifetime. No participants were excluded on the basis of TBI severity; descriptive statistics for TBI are reported in Table 1.

***Clinician-administered PTSD scale for DSM-IV (CAPS)***. The CAPS for DSM-IV (Blake et al., 1995) is a 30-item structured diagnostic interview designed to evaluate the 17 PTSD symptoms defined in the *DSM-IV* (American Psychiatric Association [APA], 1994). A continuous current (i.e. past month) PTSD severity score was calculated by summing the frequency and intensity ratings for all 17 symptoms; possible total CAPS severity scores range from 0 to 136 with higher scores representing more severe PTSD symptoms. Current PTSD diagnostic status was determined on the CAPS per *DSM-IV* (APA, 1994) criteria, with symptom frequency scores ≥ 1 and symptom intensity scores ≥ 2 required to consider a given symptom present (Blake et al., 1995). The measure was administered by PhD-level psychologists and masters-level clinicians. Both PTSD severity and diagnosis variables used in these analyses were based on current (past month) symptoms. Interrater reliability of the CAPS diagnostic determinations was K = .68, and intraclass correlation for PTSD symptom severity was *r* = .92, based on independent review of the audio tapes of 23 CAPS interviews. The study began prior to the adoption of the DSM-5, and thus CAPS-5 data were unavailable for a large subset of the sample. Therefore, we utilized the CAPS for DSM-IV to preserve sample size, as this version of the CAPS was available for all participants. Analyses focused on the continuous PTSD severity score instead of the dichotomous diagnosis because treating PTSD as a dimensional construct retains more information, statistical power, and construct validity. Dichotomizing continuous measures has been shown to lead to loss of information and increases risk for false positive results, among other concerns (e.g. Altman & Royston, 2006; Royston et al., 2006).

***Neuropsychological factor scores***. A neuropsychological test battery, comprised of tests across multiple domains (e.g. attention, memory, information processing speed, executive functioning, etc.), was administered by a BA-level technician. Given the large number of tests and subtests, we utilized factor scores that were previously generated in the same cohort to represent broader domains of neurocognitive functioning. These were derived from a confirmatory factor analysis as previously described by Wolf et al. (2023). These factor scores excluded participants who failed the Medical Symptom Validity Test (MSVT; a test of effort; Green, 2003). The factor scores captured performance on working memory, explicit memory, and attention/executive functioning. Details concerning the models are available in Wolf et al. (2023) and are only briefly described here. The Working Memory factor was indicated by raw scores on the Wechsler Adult Intelligence Scale (WAIS-IV; Wechsler, 2008) digit span forward (DSF), WAIS-IV digit span backward (DSB), and Auditory Consonant Trigrams (ACT; Stuss et al., 1985) total (0-36 s delay) tasks. Higher scores on the Working Memory factor indicated better performance. The Executive/Attentional factor was indicated by raw scores on Delis-Kaplan Executive Function System (DKEFS; Delis et al., 2001) letter fluency, category fluency, category switching, trails making (i.e. number sequencing total time, number/letter switching total time, letter sequencing total time), and colour-word interference (i.e. colour naming, word reading, inhibition, and inhibition/switching total time) tasks, the WAIS-IV digit symbol task, and the Test of Variable Attention (TOVA) D prime (Henry, 2005) task. Higher scores on the Executive/Attentional functioning factor indicated worse (slower) performance. The Explicit Memory factor was indicated by raw scores on the California Verbal Learning Test 2nd edition (CVLT; Delis et al., 2000; i.e. total trial 1–5 score, short delay free recall, long delay free recall, recognition hits) and Brief Visuospatial Memory Test-Revised (BVMT; R. Benedict, 1997) total recall and delayed recall tasks. Higher scores on the Explicit Memory factor indicated better performance. Individual-level factor scores were saved for use in analyses.

***Pittsburgh sleep quality index (PSQI)***. The PSQI (Buysse et al., 1989) is a 19-item self-report measure that assesses current (past month) sleep quality across seven domains (e.g. subjective sleep quality, sleep duration, specific sleep disturbances), which are summed and weighted equally in the calculation of a global sleep quality score (Buysse et al., 1989). Scores on the PSQI range from 0-21, with higher scores indicating worse sleep quality. We examined this global sleep quality score in our analyses. Prior evaluation of the psychometric characteristics of the PSQI global score among healthy adult controls and an adult clinical sample (depressed or sleep-disorder patients) reported strong test-retest reliability (*r* = 0.85) (Buysse et al., 1989). Global scores >5 evidenced diagnostic sensitivity and specificity of 89.6% and 86.5% respectively in distinguishing control and clinical samples (Buysse et al., 1989).

***Short-form McGill pain questionnaire (SF-MPQ)***. The SF-MPQ (Melzack, 1987) is a 15-item self-report measure, derived from the 78-item McGill Pain Questionnaire (Melzack, 1975). The SF-MPQ assesses the intensity of current (past 30 days) pain across 11 sensory and 4 affective descriptors (e.g. throbbing, shooting, stabbing), using a scale of 0–3 (Hawker et al., 2011). Total specific pain is calculated by summing the 15-item intensity scores, to reflect a total pain score ranging from 0 to 45, where higher scores indicate worse pain. The SF-MPQ also includes two additional items to assess present pain intensity (scored 0–5) and a visual analog scale for average pain (scored 0–10) (Hawker et al., 2011). We utilized the total specific pain score in our analyses, since it assesses pain severity over the past month across a wide range of pain types. Scores on the SF-MPQ are very highly correlated with scores on the long form MPQ (Melzack, 1987). Test-retest reliability for the SF-MPQ varies greatly by pain cohorts (i.e. rheumatoid arthritis vs. fibromyalgia patients) (Hawker et al., 2011). In this study, one site discontinued this measure, resulting in missing data for 174 participants.

***WHO disability assessment schedule 2.0 (WHODAS 2.0)***. The WHODAS 2.0 (Üstün et al., 2010b) is a 36-item self-report inventory that measures functional difficulty in six domains: ‘cognition, mobility, self-care, getting along, life activities (at home and at work), and social participation’ (Federici et al., 2017). Each item is ranked on a scale of 0 (none) to 4 (extreme/cannot do) and summed to create a final score between 0 and 144, where each subscale is weighted equally (Üstün et al., 2010b). These sums are then converted into percentage scores. Higher scores indicate worse functional outcomes. Prior evaluation of the psychometric characteristics of the WHODAS 2.0 cross-culturally reported strong test-retest reliability and good concurrent validity and sensitivity to change (Üstün et al., 2010a).

### 2.3. Statistical analyses

We first estimated the proportion of the cohort with current tinnitus and then examined baseline associations between tinnitus and demographic and military characteristics (age, sex, education, military branch), neuro/psychiatric diagnoses (PTSD, TBI, alcohol use disorder, and major depressive disorder), and PTSD symptom severity using chi-square analyses and *t*-tests as appropriate. We also examined the consistency of tinnitus reports over time by comparing self-reported tinnitus across T1 and T2.

Given substantial comorbidity between tinnitus, PTSD, and TBI, we compared the relative associations between each condition and a variety of cross-sectional outcomes including neuropsychological performance (in the domains of explicit memory, working memory, executive/attentional functioning), sleep disturbance, pain severity, and functional impairment. As our aim was to evaluate the extent to which tinnitus was associated with these outcomes beyond PTSD and TBI, we conducted a set of six regressions in which age and sex were entered into the first block of the model, PTSD severity in the second block, number of TBIs across the lifespan in the third block, and self-reported tinnitus in the fourth block of the model. We adjusted the *p*-value threshold across the six outcomes at the model level using the Holm multiple-testing correction procedure (Holm, 1979), such that the most stringent *p*-value threshold is adjusted by the total number of tests (k) and the next most stringent *p*-value threshold is adjusted by k-1 and so on. In other words, the most significant effect had to meet a threshold of *p* < .008 (i.e. .05/6), the second most significant effect had to meet a threshold of *p* < .01 (i.e. .05/5), etc., to be considered statistically significant. The Holm procedure reduces risk of Type II error relative to the Bonferroni method, which can be overly conservative (e.g. Perneger, 1998). Holm’s method is also more appropriate when correcting across a small number of correlated tests than is use of the False Discovery Rate (FDR; Benjamini & Hochberg, 1995). For models in which tinnitus explained significant incremental variance in a cross-sectional outcome, we conducted follow-up longitudinal models to see if T1 tinnitus was associated with changes in these outcomes over time, after first partialing out variance related to age, sex, and the T1 measurement of the T2 outcome variable (in the first step of the model), T1 PTSD (in the second step of the model), and T1 TBI (in the third step of the model). As these were models following up on multiple-testing adjusted cross-sectional models, no multiple-testing correction was applied. Additional analyses examining different definitions of TBI are described in the Supplementary Materials.

## 3. Results

### 3.1. Overlap between tinnitus, demographic characteristics, and neuro/psychiatric conditions

Descriptive characteristics for all measures are listed in Table 1. The majority of Veterans (67.5%) reported tinnitus at baseline, which was greater than the percentage of participants with clinician-diagnosed PTSD (55.9%), and less than interview-based determinations of lifetime TBI history (74.0%). Veterans with tinnitus were slightly older (*M* = 35.36 years, *SD* = 9.06) than those without (*M* = 33.39 years, *SD* = 9.01; *t* [733] = −2.764, *p* = .006). There were no significant differences in self-reports of tinnitus by sex or years of education (smallest *p* = .35). As the cohort was comprised primarily of Army and Marine Veterans, we compared the percentage of participants with tinnitus between these groups. 66.9% of Army Veterans and 75.5% of Marine Veterans reported tinnitus, and these differences were significant, χ^2^(1, *n* = 638) = 3.9, *p* = .049.

PTSD severity was significantly greater among those with (*M* = 54.95, *SD* = 28.24) versus without tinnitus (*M* = 38.0, *SD* = 26.80; *t* [733] = 7.745, *p* < .001), and tinnitus was more common among those with a current PTSD diagnosis (74.9%) than those without (58.0%), χ^2^(1, *n* = 735) = 23.6, *p* < .001. Furthermore, tinnitus was more common among those with a history of any TBI (72.6%) than those without (52.9%), χ^2^(1, *n* = 735) = 25.1, *p* < .001. The percentage of individuals with tinnitus was generally similar among those with TBI, and more common among those with vs. without TBI, regardless of the TBI definition employed, including TBIs occurring during military (80.6%), and post-military (83.9%) service periods and among those with moderate/severe TBI (87.5%), and history of blast exposure (72.0%). However, the percentage of tinnitus cases did not differ by pre-miliary TBI exposure. Additional details are provided in the Supplementary Materials.

**Figure 1.**
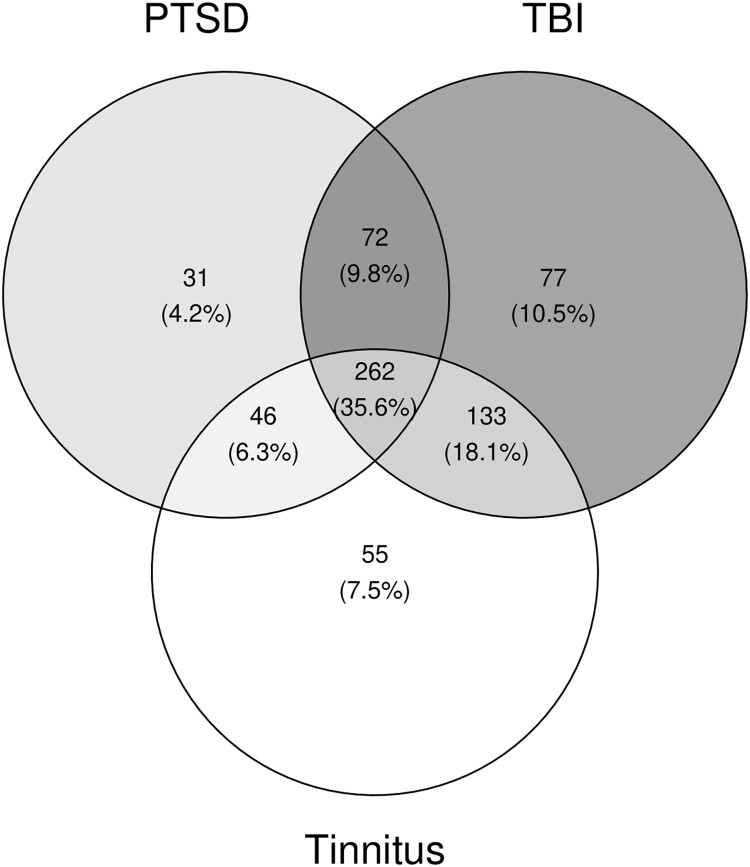
Shows the Venn diagram depicting the comorbidity between PTSD diagnosis, lifetime history of traumatic brain injury, and self-reported tinnitus at baseline. Percentages reflect the percent of the full T1 sample.

Figure 1 shows the comorbidity of PTSD, TBI, and tinnitus in the total sample: 35.6% of the full sample had all three conditions. Tinnitus was also more common among those with a current mood disorder diagnosis (77.1%) than those without (63.2%), χ^2^(1, *n* = 734) = 13.7, *p* < .001. The proportion of participants with tinnitus did not differ by alcohol use disorder (AUD) diagnosis (*p* = .630). Chi square tests revealed that among those who reported having tinnitus at T1, 94.3% reported having tinnitus at T2 (*p* < .001), and of those who reported having no tinnitus at T1, 30.2% reported new onset tinnitus at T2 (*p* < .001). 71.0% of Veterans reported tinnitus at follow-up.

### 3.2. Zero-order cross-sectional associations with tinnitus

At T1, point-biserial correlations revealed that tinnitus was associated with PTSD severity (*r* = .275, *p* < .001), number of TBIs (*r* = .155, *p* < .001), explicit memory factor scores (*r* = −.100, *p* = .007), executive/attentional factor scores (*r* = .104, *p* = .005), sleep disturbance (*r* = .244, *p* < .001), pain severity (*r* = .338, *p* < .001), and functional impairment (*r* = .327, *p* < .001). Tinnitus was not significantly correlated with working memory factor scores (*r* = −.068, *p* = .066).

### 3.3. Additional variance explained by tinnitus beyond PTSD and TBI in health outcomes

As shown in Table 2, PTSD was associated with working (β = −.226, *p* < .001) and explicit memory performance (β = −.266, *p* < .001), executive/attentional functioning (β = .242, *p* < .001), sleep disturbance (β = .626, *p* < .001), pain severity (β = .500, *p* < .001), and functional impairment (β = .657, *p* < .001), explaining an additional 5.1-43.1% of the variance in these outcomes beyond age and sex. All PTSD associations passed the multiple testing adjusted *p*-value threshold. TBI explained an additional 0.5% of the variance (*p* = .040) in explicit memory performance (β = .073, *p* = .040) beyond PTSD and an additional 0.7% of the variance (*p* = .023) in pain severity (β = .085, *p* = .023) beyond PTSD, but neither of these associations met the multiple testing adjusted *p*-value threshold.

**Table 2.** Cross-sectional health and functional outcomes of tinnitus beyond PTSD and TBI.

Outcome	β	B	SE	*p*	*ΔR^2^*	*p*
Working memory (*n* *=* 731)						
Block 1					.048	<.001
Age	−.219	−.026	.004	<.001		
Sex	.024	.088	.131	.503		
Block 2: PTSD	−.**226**	−.**008**	.**001**	**<**.**001**	.**051**	**<**.**001**
Block 3: TBI	.061	.028	.017	.094	.003	.094
Block 4: Tinnitus	.015	.034	.085	.689	<.001	.689
Explicit memory (*n* = 731)						
Block 1					.074	<.001
Age	−.263	−.016	.002	<.001		
Sex	.089	.165	.066	.013		
Block 2: PTSD	−.**266**	−.**005**	.**001**	**<**.**001**	.**071**	**<**.**001**
Block 3: TBI	.073	.017	.008	.040	.005	.040
Block 4: Tinnitus	−.004	−.004	.043	.920	<.001	.920
Executive attention (*n* = 731)				* *	* *	* *
Block 1					.062	<.001
Age	.243	.063	.009	<.001		
Sex	−.073	−.577	.286	.044		
Block 2: PTSD	.**242**	.**020**	.**003**	**<**.**001**	.**058**	**<**.**001**
Block 3: TBI	−.041	−.041	.037	.261	.002	.261
Block 4: Tinnitus	.015	.076	.185	.682	<.001	.682
Sleep disturbance (*n* *=* 697)				* *	* *	* *
Block 1					.002	.478
Age	.046	.024	.020	.225		
Sex	.001	.009	.618	.989		
Block 2: PTSD	.**626**	.**105**	.**005**	**<**.**001**	.**391**	**<**.**001**
Block 3: TBI	.022	.045	.062	.464	<.001	.464
Block 4: Tinnitus	.070	.716	.318	.025	.004	.025
Pain (*n* *=* 561)				* *	* *	* *
Block 1					.022	.002
Age	.137	.130	.040	.001		
Sex	.051	1.502	1.234	.224		
Block 2: PTSD	.**500**	.**151**	.**011**	**<**.**001**	.**250**	**<**.**001**
Block 3: TBI	.085	.301	.132	.023	.007	.023
Block 4: Tinnitus	.**184**	**3**.**383**	.**690**	**<**.**001**	.**030**	**<**.**001**
Functional impairment (*n* = 694)						
Block 1					.011	.022
Age	.105	.217	.078	.006		
Sex	−.007	−.472	2.401	.844		
Block 2: PTSD	.**657**	.**428**	.**019**	**<**.**001**	.**431**	**<**.**001**
Block 3: TBI	.012	.094	.232	.687	<.001	.687
Block 4: Tinnitus	.**145**	**5**.**765**	**1**.**172**	**<**.**001**	.**019**	**<**.**001**

Note. Significant effects that passed multiple-testing correction are bolded. PTSD = posttraumatic stress disorder; TBI = traumatic brain injury; SE = standard error; β = standardized coefficient; B = unstandardized coefficient.

After accounting for the variance explained by age, sex, PTSD, and TBI, tinnitus was associated with an additional 3.0% of the variance (*p* < .001) in pain severity (β = .184, *p* < .001), and an additional 1.9% of the variance (*p* < .001) in functional impairment (β = .145, *p* < .001). Both associations surpassed our multiple testing adjusted *p*-value thresholds. However, for the sleep disturbance model, the additional 0.4% variance explained by tinnitus did not surpass the multiple testing *p*-value adjustment (*p* = .025). Tinnitus did not contribute any further variance beyond PTSD and TBI in working memory performance, explicit memory performance, or executive/attentional functioning. Additional models examining different definitions of TBI, interactions between tinnitus and PTSD and tinnitus and TBI, and those examining tinnitus impairment scores (in a small subset of the cohort) are described in the Supplementary Materials and in Tables S1–S7; none substantively altered the primary results described in the main text.

### 3.4. Follow-up analyses: longitudinal associations with tinnitus

For cross-sectional outcomes in which tinnitus achieved multiple testing-adjusted significant associations (pain severity and functional impairment), we conducted follow-up regressions to test if T1 tinnitus predicted additional variance in T2 outcomes beyond T1 PTSD severity, T1 number of TBIs, and the T1 score on the respective T2 outcome. T1 PTSD severity explained an additional 5.4% of the variance (*p* < .001) in T2 pain severity (β = .287, *p* < .001) beyond age, sex, and T1 pain severity, and an additional 3.4% (*p* < .001) of the variance in T2 functional impairment (β = .248, *p* < .001) beyond age, sex, and T1 functional impairment. T1 TBI count did not account for additional variance beyond age, sex, and PTSD in either model. Similarly, T1 tinnitus was not associated with either T2 outcome beyond PTSD and TBI in models predicting T2 pain severity (*p* = .310) or T2 functional impairment (*p* = .822; Table 3).

**Table 3.** Longitudinal health and functional outcomes of tinnitus beyond PTSD and TBI.

Outcome	β	B	SE	*p*	*ΔR^2^*	*p*
T2 Pain (*n* *=* 338)						
Block 1					.438	<.001
Age	.061	.051	.035	.138		
Sex	.048	1.250	1.077	.247		
T1 Pain	.651	.694	.044	<.001		
Block 2: T1 PTSD	.**287**	.**081**	.**014**	**<**.**001**	.**054**	**<**.**001**
Block 3: T1 TBI	.023	.068	.122	.576	.000	.576
Block 4: T1 Tinnitus	.044	.741	.729	.310	.002	.310
T2 Functional impairment (*n* = 421)						
Block 1					.511	<.001
Age	−.027	−.046	.059	.435		
Sex	.012	.638	1.846	.730		
T1 WHODAS	.717	.694	.033	<.001		
Block 2: T1 PTSD	.**248**	.**145**	.**026**	**<**.**001**	.**034**	**<**.**001**
Block 3: T1 TBI	−.045	−.282	.215	.190	.002	.190
Block 4: T1 Tinnitus	−.008	−.276	1.227	.822	.000	.822

Note. Significant effects that passed multiple-testing correction are bolded. PTSD = posttraumatic stress disorder; TBI = traumatic brain injury; T1 = time 1; WHODAS = World Health Organization Disability Assessment Schedule 2.0; SE = standard error; β = standardized coefficient; B = unstandardized coefficient.

## 4. Discussion

This is the largest published study to date to examine comorbidity between tinnitus, PTSD, and TBI and their relative relationships with other health and functional outcomes in Veterans. Results help to characterize the nature of tinnitus comorbidity and identify unique associations with pain and functional impairment in a sample of post-9/11 veterans. We found that over two-thirds of this cohort reported tinnitus (67.5%). Our estimate of tinnitus in this cohort replicated that from some smaller prior studies of post-9/11 Veterans (63-67%; Henry et al., 2019, 2021). However, other studies of post-9/11 Veterans have reported much lower estimates of tinnitus presence (∼13-15%) when using the VA electronic health record (EHR) and International Classification of Diseases (ICD) codes to identify the condition (Martz et al., 2018; Swan et al., 2017). This may suggest that tinnitus is not systematically assessed in VA clinical care or that estimates are lower in epidemiological cohorts versus clinical samples. That the vast majority of post-9/11 Veterans in this study reported tinnitus is clinically significant, given that these young Veterans may experience chronic tinnitus and associated impairment for decades to come.

In this study, over one-third of the cohort had tinnitus with comorbid PTSD and TBI. No other published study to date has reported on the overlap of all three conditions, but many studies have evaluated pairwise relationships between two of these conditions. Our results broadly replicate prior ones which have suggested that tinnitus frequently co-occurs with PTSD (55% per Terhaag et al., 2021) and TBI (34-59% per Karch et al., 2016; Knoll et al., 2020; Le et al., 2024; MacGregor et al., 2020). This comorbidity means it is important to differentiate the effects of tinnitus on other health outcomes from PTSD and TBI, as this can influence which condition requires the most intense or immediate clinical attention.

PTSD predicted poorer outcomes in all domains, adding to the vast literature that supports associations between PTSD and poorer physical, cognitive, and functional health (Benedict et al., 2020; Goldberg et al., 2014; Lawrence et al., 2023; Lind et al., 2017). The number of TBIs sustained across the lifetime did not predict poorer outcomes in any domain beyond PTSD. Tinnitus predicted worse concurrent pain severity, beyond PTSD and TBI, replicating prior evidence of an association between tinnitus and worse pain outcomes (Ausland et al., 2021). Likewise, tinnitus predicted worse concurrent functional impairment beyond PTSD and TBI, expanding on literature showing that scores on functional impairment measures (e.g. WHODAS-2.0) differ significantly between tinnitus and non-tinnitus groups (Henry et al., 2019). Thus, while tinnitus is highly comorbid with PTSD and TBI, it is important to consider its unique role in adverse health outcomes when providing clinical care and conducting research among Veterans with PTSD and/or TBI.

Tinnitus did not explain additional variance in models predicting explicit memory or executive/attentional functioning, and we saw no correlation between tinnitus and working memory. The literature on these associations is equivocal, with some prior studies suggesting tinnitus was related to memory and attention (Clarke et al., 2020; Kuchinsky et al., 2020; Rossiter et al., 2006) and others finding more limited or no evidence of an association (Mohamad et al., 2016). This cohort is relatively young, and it is possible that cognitive performance effects may only become evident at older ages, due to greater variability in cognitive performance at older ages (Christensen et al., 1999; Yang et al., 2024). Tinnitus also did not further explain sleep disturbance beyond PTSD and TBI.

Tinnitus did not predict worsening pain or functional impairment over time after accounting for baseline pain/impairment and PTSD and TBI. One possible explanation for differences in cross-sectional versus longitudinal results is that the study lacked sufficient power to detect longitudinal associations because the follow-up cohort was smaller. The factors influencing changes in pain and functional impairment over time may also differ from those associated with current levels. These results suggest it may be the chronic daily PTSD symptoms that are most related to adverse outcomes over time, which highlights the importance of including PTSD in studies evaluating TBI and tinnitus.

## 5. Clinical implications

Taken together, results carry implications for clinical assessment and treatment of these common comorbidities among Veterans. Clinicians might consider evaluating all three conditions when patients present with symptoms of one. Stress is known to make tinnitus worse (Baigi et al., 2011; Kim et al., 2015; Moring et al., 2022b), particularly in cases where tinnitus symptoms are associated with trauma (Fagelson, 2022; Hinton et al., 2006). Thus, clinicians may wish to assess tinnitus during PTSD treatment, as reducing stress through the course of PTSD treatment may have secondary positive effects on tinnitus symptom severity (Moring et al., 2022a). In turn, this may minimize the effects of tinnitus on other health and functional outcomes. Likewise, successful tinnitus management could potentially have generalizing positive effects on mitigating PTSD-associated pain and functional impairment.

## 6. Future directions

An important goal for future tinnitus research is to improve the measurement of the condition and use a shared definition across research studies. Increasing measurement consistency will facilitate comparisons across studies. Future work could also examine how tinnitus changes in response to treatment for related conditions such as PTSD; this is another important area of research that requires the use of high-quality measures that are sensitive to change, such as the Tinnitus Functional Index (TFI; Langguth & Gilles, 2024; Meikle et al., 2012). It will also be important for future tinnitus assessment research to consider the potential role of over-reporting of tinnitus symptoms (e.g. due to generalized distress or secondary gain) and distinguish this from true tinnitus cases.

## 7. Limitations

Several study limitations should be considered when evaluating these results. Tinnitus presence was assessed using a single item self-report measure, which may be unreliable and does not capture severity or functional impact. While other studies also rely on self-reported single-item measures (Biswas, et al., 2023; Le et al., 2024; McCormack et al., 2016), this does not substitute for a validated and comprehensive measure of tinnitus presence, severity, and impact such as the THI. This concern is offset, to an extent, by our follow-up analyses in a small subset of this cohort with the THI, which showed results that replicated those using the single-item measure (see Supplementary Materials). Still, this underscores the need for the development of standardized approaches to assessing and diagnosing tinnitus, for which there is currently no standard as most medical record diagnoses of tinnitus are based on patient self-report. Related to this, we did not account for hearing loss, as we did not have this information for most of the cohort (a hearing test was added later to the protocol and thus was missing for 80% of the sample). It is unknown if these results generalize to other populations, given that our cohort was largely white, male, and young post-9/11 Veterans and not nationally representative of all US Veterans. As tinnitus becomes more common and more severe with age (Bhatt et al., 2016; Reisinger et al., 2023), it is important to expand future research in this area to include older Veterans. Finally, we examined a *DSM-IV* measure of PTSD symptom severity to maximize sample size in this cohort and it is unknown if results generalize to *DSM-5* measures of PTSD. This concern is offset by evidence that total scores on the CAPS for *DSM-IV* correlate strongly with total scores on the CAPS-5 (Weathers et al., 2018).

## 8. Conclusions

Tinnitus is very common among young post-9/11 Veterans and highly comorbid with PTSD and TBI. Tinnitus was associated with worse pain and functional impairment beyond these other conditions. The high proportion of tinnitus cases in such a young population is concerning, given that there are limited treatments for tinnitus and that the condition tends to be chronic. This means that Veterans may have many years of tinnitus-related distress, and it underscores the importance of identifying these Veterans and developing effective therapies. This may, in turn, minimize the collective health impact of these conditions and promote greater functioning and wellness.

## Supplementary Material

Final Supplemental Materials.docx

## Data Availability

Qualified investigators may write to the TRACTS data repository (catherine.fortier@va.gov) to apply to access data.

## Note

1 The current study added a measure of tinnitus impact (as opposed to tinnitus presence) called the Tinnitus Handicap Inventory (THI; Newman et al., 1996, 1998) much later in the study protocol and thus data on this measure was missing for ∼77% (*n* = 568) of participants, which precluded its evaluation in the main analyses of this study. In the small subset of participants with this measure, self-reported tinnitus presence correlated with THI total score at *r* = .50, *p* < .001. Additional analyses involving the THI are presented in Tables S1 and S2 of the Supplementary Materials.
