# Supplementary material for: Tinnitus among post-9/11 veterans: psychiatric comorbidity and associations with health and functional outcomes
Source: Eur J Psychotraumatol. 2026 Feb 26;17(1):2623718. doi: 10.1080/20008066.2026.2623718 (PMC12947285; doi:10.1080/20008066.2026.2623718)
Supplement: Final Supplemental Materials.docx [file ZEPT_A_2623718_SM1708.docx]

Tinnitus Among Post-9/11 Veterans: Psychiatric Comorbidity and Associations with Health and Functional Outcomes

**Supplementary Materials**

**Additional Variance Explained by Tinnitus Handicap Beyond PTSD and TBI in Health Outcomes**

We replicated the structure of the models in Table 2, replacing the dichotomous tinnitus variable with Tinnitus Handicap Inventory (THI; Newman et al., 1996) total scores to assess how much of the variance tinnitus handicap explained beyond the effects of PTSD severity and number of lifetime TBIs. These findings are presented in Table S1. THI data was only available in *n* = 167 participants at T1.

As shown in Table S1, PTSD predicted worse sleep disturbance, worse pain, and worse functional impairment, and all three of these effects withstood multiple-testing correction (all *p*s <.001). TBI nominally predicted worse sleep disturbance beyond PTSD, but this effect did not pass multiple-testing correction (*p* = .030). Consistent with the results presented in the main text, tinnitus handicap total scores predicted worse pain (β = .32, *p* < .001, *R*^2^ change = 6.8%) and functional impairment (β = .24, *p* < .001, *R*^2^ change = 3.8%) beyond both PTSD and TBI, and both effects passed multiple-testing correction.

Associations between PTSD and working memory, explicit memory, and executive attention that were observed in the larger cohort were not significant in these supplemental analyses. This may be related to the smaller sample size and associated reduced statistical power. As with our primary models presented in the main text, there were no significant TBI associations. In sum, these results for tinnitus impairment paralleled those for presence/absence of tinnitus in the larger cohort.

**Longitudinal Associations with Tinnitus Handicap Total Scores**

We also replicated the longitudinal models in Table 3, replacing the dichotomous tinnitus variable with THI total scores to assess the variance explained by T1 tinnitus handicap in predicting residualized change in pain and functional impairment at T2. These analyses are presented in Table S2. As with results described in the main text, tinnitus handicap total scores at T1 did not predict T2 health outcomes in pain or functional impairment. PTSD remained significantly associated with these two health outcomes at T2. These results are also essentially unchanged from those evaluated in the larger cohort at in the main text.

**Addition of Education as a Covariate in Models Evaluating Neurocognitive Outcomes**

We replicated the models in the main text (Table 2) but added education to the first block of the model. Education was associated with each factor score: working memory (β = .209, *p* < .001), explicit memory (β = .186, *p* < .001), and executive/attention (β = -.180, *p* < .001). However, covarying for education did not change the pattern of results for tinnitus (all null), nor did it change the significance or direction of the significant PTSD effects (Table S3).

**Additional Variance Explained by TBI Burden Scores and Blast Exposure**

We exchanged number of lifetime TBIs in our primary models with the TBI Lifetime Burden score on the Boston Assessment of TBI. This total score represents both number and severity of TBIs across the lifespan. Our primary results for tinnitus were essentially unchanged (Table S4). The TBI burden variable explained additional variance beyond PTSD and demographics in pain severity (β = .12, *p* = .001, *R*^2^ change = 1.4%) only. This effect passed multiple testing correction, however, tinnitus still explained significant additional variance in pain severity (β = .18, *p* < .001, *R*^2^ change = 2.8%) beyond lifetime TBI burden. Additionally, tinnitus still explained significant additional variance in functional impairment (β = .14, *p* < .001, *R*^2^ change = 1.8%) beyond lifetime TBI burden.

History of blast exposures did not significantly explain any additional variance in any of the models, and tinnitus continued to predict additional variance in the pain (β = .19, *p* < .001, *R*^2^ change = 3.3%) and functional impairment (β = .14, *p* < .001, *R*^2^ change = 1.9%) models. Results are detailed in Table S5.

**Interaction Terms**

We evaluated if tinnitus and PTSD or tinnitus and number of lifetime TBIs interacted with each other in association with each health outcome variable. To do so, we added an extra step to our models to include tinnitus x PTSD, and separately, tinnitus x TBI. The Holm multiple testing correction was again applied across the 12 new interaction term models (smallest adjusted *p*-value threshold: *p* < .004) We found that no interaction terms surpassed this multiple testing correction threshold. There were no nominally significant interactions involving tinnitus X number of lifetime TBIs. There were two nominally significant interactions involving tinnitus X PTSD severity in relationship to functional impairment (β = .20, *p* = .007), and pain (β = .22, *p* = .024). Further details can be found in Tables S6 and S7.

**TBI Definition Comparisons**

We conducted a series of chi-square analyses to examine whether self-reported tinnitus presence differed by TBI timing (pre-military, military, and post-military service periods) and by additional TBI definitions (moderate/severe TBI and blast exposure). The percentage of participants who reported tinnitus did not meaningfully differ between those with (64.2%) versus without (69.6%) a history of pre-military TBI (χ^2^ = 2.3, *p* = .125). However, tinnitus was more common among those with military-related TBIs (80.6% vs. 52.1%; χ^2^ = 66.9, *p* < .001), and post-military TBIs (83.9% vs. 66.2%; χ^2^ = 7.4, *p* = .006), and among those with a history of blast exposure (72.0% vs. 47.0%; χ^2^ = 30.85, *p* < .001), and among those with moderate/severe TBI (87.5% vs. 66.6%; χ^2^ = 6.09, *p* = .014). The proportion of Veterans reporting tinnitus among each definition of TBI was also similar across these TBI definitions (ranging from 64.2 – 87.5%). The percentage of individuals with TBI as a function of tinnitus presence (i.e., the other direction of analysis) is reported in Table 1.

**Table S1**

*Cross-Sectional Health and Functional Outcomes of Tinnitus Handicap Beyond PTSD and TBI*

| Outcome | β | B | SE | *p* | *ΔR^2^* | *p* |
| --- | --- | --- | --- | --- | --- | --- |
| Working Memory (*n =* 167) |  |  |  |  |  |  |
| Block 1 |  |  |  |  | .080 | .001 |
| Age | -.250 | -.027 | .008 | .001 |  |  |
| Sex | .097 | .324 | .253 | .202 |  |  |
| Block 2: PTSD | -.115 | -.004 | .003 | .127 | .013 | .127 |
| Block 3: TBI | .131 | .041 | .024 | .093 | .016 | .093 |
| Block 4: THI | -.101 | -.005 | .004 | .260 | .007 | .260 |
| Explicit Memory (*n* = 167) |  |  |  |  |  |  |
| Block 1 |  |  |  |  | .132 | <.001 |
| Age | -.271 | -.015 | .004 | <.001 |  |  |
| Sex | .202 | .348 | .127 | .007 |  |  |
| Block 2: PTSD | -.102 | -.002 | .001 | .161 | .010 | .161 |
| Block 3: TBI | .097 | .016 | .012 | .202 | .009 | .202 |
| Block 4: THI | -.037 | -.001 | .002 | .668 | .001 | .668 |
| Executive Attention (*n* = 167) |  |  |  |  |  |  |
| Block 1 |  |  |  |  | .134 | <.001 |
| Age | .302 | .063 | .015 | <.001 |  |  |
| Sex | -.163 | -1.081 | .487 | .028 |  |  |
| Block 2: PTSD | .127 | .009 | .005 | .081 | .016 | .081 |
| Block 3: TBI | -.062 | -.039 | .047 | .414 | .004 | .414 |
| Block 4: THI | .163 | .015 | .008 | .060 | .018 | .060 |
| Sleep Disturbance (*n =* 159) |  |  |  |  |  |  |
| Block 1 |  |  |  |  | .008 | .515 |
| Age | .060 | .029 | .039 | .456 |  |  |
| Sex | -.060 | -.899 | 1.209 | .458 |  |  |
| Block 2: PTSD | **.606** | **.100** | **.010** | **<.001** | **.367** | **<.001** |
| Block 3: TBI | -.144 | -.204 | .093 | .030 | .019 | .030 |
| Block 4: THI | .119 | .026 | .016 | .118 | .010 | .118 |
| Pain (*n =* 165) |  |  |  |  |  |  |
| Block 1 |  |  |  |  | .074 | .002 |
| Age | .221 | .200 | .069 | .004 |  |  |
| Sex | .199 | 5.620 | 2.165 | .010 |  |  |
| Block 2: PTSD | **.494** | **.152** | **.020** | **<.001** | **.244** | **<.001** |
| Block 3: TBI | .088 | .235 | .181 | .198 | .007 | .198 |
| Block 4: THI | **.315** | **.127** | **.030** | **<.001** | **.068** | **<.001** |
| Functional impairment (*n* = 167) |  |  |  |  |  |  |
| Block 1 |  |  |  |  | .016 | .258 |
| Age | .130 | .219 | .133 | .100 |  |  |
| Sex | .020 | 1.082 | 4.173 | .796 |  |  |
| Block 2: PTSD | **.657** | **.379** | **.034** | **<.001** | **.431** | **<.001** |
| Block 3: TBI | -.055 | -.275 | .306 | .370 | .003 | .370 |
| Block 4: THI | **.235** | **.178** | **.051** | **<.001** | **.038** | **<.001** |

*Note*. Significant effects that passed multiple-testing correction are bolded. PTSD = posttraumatic stress disorder; TBI = traumatic brain injury; THI = Tinnitus Handicap Inventory; SE = standard error; β = standardized coefficient; B = unstandardized coefficient

**Table S2.**

*Longitudinal Health and Functional Outcomes of Tinnitus Handicap Beyond PTSD and TBI*

| Outcome | β | B | SE | *p* | *ΔR^2^* | *p* |
| --- | --- | --- | --- | --- | --- | --- |
| T2 Pain (*n =* 104) |  |  |  |  |  |  |
| Block 1 |  |  |  |  | .408 | <.001 |
| Age | .087 | .069 | .064 | .286 |  |  |
| Sex | .077 | 1.960 | 1.992 | .327 |  |  |
| T1 Pain | .606 | .645 | .085 | <.001 |  |  |
| Block 2: T1 PTSD | **.340** | **.091** | **.024** | **<.001** | **.077** | **<.001** |
| Block 3: T1 TBI | .054 | .112 | .160 | .485 | .003 | .485 |
| Block 4: T1 THI | -.024 | -.010 | .036 | .789 | .000 | .789 |
| T2 Functional impairment (*n* = 105) |  |  |  |  |  |  |
| Block 1 |  |  |  |  | .538 | <.001 |
| Age | .016 | .025 | .109 | .815 |  |  |
| Sex | -.034 | -1.694 | 3.485 | .628 |  |  |
| T1 WHODAS | .728 | .724 | .068 | <.001 |  |  |
| Block 2: T1 PTSD | **.249** | **.133** | **.050** | **.009** | **.031** | **.009** |
| Block 3: T1 TBI | -.015 | -.064 | .294 | .827 | .000 | .827 |
| Block 4: T1 THI | -.054 | -.043 | .065 | .510 | .002 | .510 |

*Note*. Significant effects that passed multiple-testing correction are bolded. PTSD = posttraumatic stress disorder; TBI = traumatic brain injury; THI = Tinnitus Handicap Inventory; T1 = time 1; WHODAS = World Health Organization Disability Assessment Schedule 2.0; SE = standard error; β = standardized coefficient; B = unstandardized coefficient.

**Table S3**

*Cross-Sectional Neurocognitive Outcomes of Tinnitus Beyond PTSD and TBI with Education Included (n = 731)*

| Outcome | β | B | SE | *p* | *ΔR^2^* | *p* |
| --- | --- | --- | --- | --- | --- | --- |
| Working Memory |  |  |  |  |  |  |
| Block 1 |  |  |  |  | **.087** | **<.001** |
| Age | **-.282** | **-.033** | **.004** | **<.001** |  |  |
| Sex | .003 | .012 | .129 | .924 |  |  |
| Education | **.209** | **.105** | **.019** | **<.001** |  |  |
| Block 2: PTSD | **-.196** | **-.007** | **.001** | **<.001** | **.037** | **<.001** |
| Block 3: TBI | .049 | .022 | .017 | .177 | .002 | .177 |
| Block 4: Tinnitus | .019 | .042 | .084 | .613 | .000 | .613 |
| Explicit Memory |  |  |  |  |  |  |
| Block 1 |  |  |  |  | **.105** | **<.001** |
| Age | **-.319** | **-.019** | **.002** | **<.001** |  |  |
| Sex | .070 | .131 | .066 | .047 |  |  |
| Education | **.186** | **.048** | **.010** | **<.001** |  |  |
| Block 2: PTSD | **-.242** | **-.005** | **.001** | **<.001** | **.056** | **<.001** |
| Block 3: TBI | .063 | .015 | .008 | .075 | .004 | .075 |
| Block 4: Tinnitus | -.001 | -.001 | .042 | .984 | .000 | .984 |
| Executive Attention |  |  |  |  |  |  |
| Block 1 |  |  |  |  | **.091** | **<.001** |
| Age | **.298** | **.078** | **.010** | **<.001** |  |  |
| Sex | -.055 | -.434 | .283 | .126 |  |  |
| Education | **-.180** | **-.200** | **.041** | **<.001** |  |  |
| Block 2: PTSD | **.218** | **.018** | **.003** | **<.001** | **.046** | **<.001** |
| Block 3: TBI | -.030 | -.031 | .036 | .396 | .001 | .396 |
| Block 4: Tinnitus | .012 | .061 | .184 | .741 | .000 | .741 |

*Note*. Significant effects that passed multiple-testing correction are bolded. PTSD = posttraumatic stress disorder; TBI = traumatic brain injury; SE = standard error; β = standardized coefficient; B = unstandardized coefficient

**Table S4**

*Cross-Sectional Health and Functional Outcomes of Tinnitus Beyond PTSD and Lifetime TBI Burden*

| Outcome | β | B | SE | *p* | *ΔR^2^* | *p* |
| --- | --- | --- | --- | --- | --- | --- |
| Working Memory (*n =* 731) |  |  |  |  |  |  |
| Block 1 |  |  |  |  | .048 | <.001 |
| Age | -.219 | -.026 | .004 | <.001 |  |  |
| Sex | .024 | .088 | .131 | .503 |  |  |
| Block 2: PTSD | **-.226** | **-.008** | **.001** | **<.001** | **.051** | **<.001** |
| Block 3: TBI Burden | .017 | .005 | .011 | .649 | .000 | .649 |
| Block 4: Tinnitus | .019 | .043 | .085 | .617 | .000 | .617 |
| Explicit Memory (*n* = 731) |  |  |  |  |  |  |
| Block 1 |  |  |  |  | .074 | <.001 |
| Age | -.263 | -.016 | .002 | <.001 |  |  |
| Sex | .089 | .165 | .066 | .013 |  |  |
| Block 2: PTSD | **-.266** | **-.005** | **.001** | **<.001** | **.071** | **<.001** |
| Block 3: TBI Burden | .029 | .005 | .006 | .421 | .001 | .421 |
| Block 4: Tinnitus | .000 | .000 | .043 | .992 | .000 | .992 |
| Executive Attention (*n* = 731) |  |  |  |  |  |  |
| Block 1 |  |  |  |  | .062 | <.001 |
| Age | .243 | .063 | .009 | <.001 |  |  |
| Sex | -.073 | -.577 | .286 | .044 |  |  |
| Block 2: PTSD | **.242** | **.020** | **.003** | **<.001** | **.058** | **<.001** |
| Block 3: TBI Burden | .005 | .003 | .025 | .891 | .000 | .891 |
| Block 4: Tinnitus | .011 | .054 | .186 | .772 | .000 | .772 |
| Sleep Disturbance (*n =* 697) |  |  |  |  |  |  |
| Block 1 |  |  |  |  | .002 | .478 |
| Age | .046 | .024 | .020 | .225 |  |  |
| Sex | .001 | .009 | .618 | .989 |  |  |
| Block 2: PTSD | **.626** | **.105** | **.005** | **<.001** | **.391** | **<.001** |
| Block 3: TBI Burden | .038 | .052 | .042 | .215 | .001 | .215 |
| Block 4: Tinnitus | .068 | .693 | .318 | .030 | .004 | .030 |
| Pain (*n =* 561) |  |  |  |  |  |  |
| Block 1 |  |  |  |  | .022 | .002 |
| Age | .137 | .130 | .040 | .001 |  |  |
| Sex | .051 | 1.502 | 1.234 | .224 |  |  |
| Block 2: PTSD | **.500** | **.151** | **.011** | **<.001** | **.250** | **<.001** |
| Block 3: TBI Burden | **.121** | **.293** | **.090** | **.001** | **.014** | **.001** |
| Block 4: Tinnitus | **.178** | **3.272** | **.690** | **<.001** | **.028** | **<.001** |
| Functional impairment (*n* = 694) |  |  |  |  |  |  |
| Block 1 |  |  |  |  | .011 | .022 |
| Age | .105 | .217 | .078 | .006 |  |  |
| Sex | -.007 | -.472 | 2.401 | .844 |  |  |
| Block 2: PTSD | **.657** | **.428** | **.019** | **<.001** | **.431** | **<.001** |
| Block 3: TBI Burden | .034 | .178 | .157 | .257 | .001 | .257 |
| Block 4: Tinnitus | **.143** | **5.677** | **1.175** | **<.001** | **.018** | **<.001** |

*Note*. Significant effects that passed multiple-testing correction are bolded. PTSD = posttraumatic stress disorder; TBI = traumatic brain injury; SE = standard error; β = standardized coefficient; B = unstandardized coefficient

**Table S5**

*Cross-Sectional Health and Functional Outcomes of Tinnitus Beyond PTSD and History of Blast Exposure*

| Outcome | β | B | SE | *p* | *ΔR^2^* | *p* |
| --- | --- | --- | --- | --- | --- | --- |
| Working Memory (*n =* 731) |  |  |  |  |  |  |
| Block 1 |  |  |  |  | .048 | <.001 |
| Age | -.219 | -.026 | .004 | <.001 |  |  |
| Sex | .024 | .088 | .131 | .503 |  |  |
| Block 2: PTSD | **-.226** | **-.008** | **.001** | **<.001** | **.051** | **<.001** |
| Block 3: Blast | -.051 | -.142 | .102 | .164 | .002 | .164 |
| Block 4: Tinnitus | .028 | .065 | .086 | .446 | .001 | .446 |
| Explicit Memory (*n* = 731) |  |  |  |  |  |  |
| Block 1 |  |  |  |  | .074 | <.001 |
| Age | -.263 | -.016 | .002 | <.001 |  |  |
| Sex | .089 | .165 | .066 | .013 |  |  |
| Block 2: PTSD | **-.266** | **-.005** | **.001** | **<.001** | **.071** | **<.001** |
| Block 3: Blast | -.038 | -.055 | .051 | .282 | .001 | .282 |
| Block 4: Tinnitus | .009 | .011 | .043 | .806 | .000 | .806 |
| Executive Attention (*n* = 731) |  |  |  |  |  |  |
| Block 1 |  |  |  |  | .062 | <.001 |
| Age | .243 | .063 | .009 | <.001 |  |  |
| Sex | -.073 | -.577 | .286 | .044 |  |  |
| Block 2: PTSD | **.242** | **.020** | **.003** | **<.001** | **.058** | **<.001** |
| Block 3: Blast | .053 | .326 | .222 | .143 | .003 | .143 |
| Block 4: Tinnitus | .003 | .017 | .186 | .926 | .000 | .926 |
| Sleep Disturbance (*n =* 697) |  |  |  |  |  |  |
| Block 1 |  |  |  |  | .002 | .478 |
| Age | .046 | .024 | .020 | .225 |  |  |
| Sex | .001 | .009 | .618 | .989 |  |  |
| Block 2: PTSD | **.626** | **.105** | **.005** | **<.001** | **.391** | **<.001** |
| Block 3: Blast | .070 | .882 | .383 | .022 | .005 | .022 |
| Block 4: Tinnitus | .062 | .634 | .319 | .047 | .003 | .047 |
| Pain (*n =* 561) |  |  |  |  |  |  |
| Block 1 |  |  |  |  | .022 | .002 |
| Age | .137 | .130 | .040 | .001 |  |  |
| Sex | .051 | 1.502 | 1.234 | .224 |  |  |
| Block 2: PTSD | **.500** | **.151** | **.011** | **<.001** | **.250** | **<.001** |
| Block 3: Blast | .014 | .309 | .847 | .715 | .000 | .715 |
| Block 4: Tinnitus | **.193** | **3.550** | **.696** | **<.001** | **.033** | **<.001** |
| Functional impairment (*n* = 694) |  |  |  |  |  |  |
| Block 1 |  |  |  |  | .011 | .022 |
| Age | .105 | .217 | .078 | .006 |  |  |
| Sex | -.007 | -.472 | 2.401 | .844 |  |  |
| Block 2: PTSD | **.657** | **.428** | **.019** | **<.001** | **.431** | **<.001** |
| Block 3: Blast | .024 | 1.146 | 1.430 | .423 | .001 | .423 |
| Block 4: Tinnitus | **.144** | **5.744** | **1.179** | **<.001** | **.019** | **<.001** |

*Note*. Significant effects that passed multiple-testing correction are bolded. Hx = history; PTSD = posttraumatic stress disorder; Blast = number of military blast exposures; SE = standard error; β = standardized coefficient; B = unstandardized coefficient

**Table S6**

*Tinnitus X PTSD in Predicting Cross-Sectional Health and Functional Outcomes*

| Outcome | β | B | SE | *p* | *ΔR^2^* | *p* |
| --- | --- | --- | --- | --- | --- | --- |
| Working Memory (*n =* 731) |  |  |  |  |  |  |
| Block 1 |  |  |  |  | .048 | <.001 |
| Age | -.219 | -.026 | .004 | <.001 |  |  |
| Sex | .024 | .088 | .131 | .503 |  |  |
| Block 2: PTSD | **-.226** | **-.008** | **.001** | **<.001** | **.051** | **<.001** |
| Block 3: TBI | .061 | .028 | .017 | .094 | .003 | .094 |
| Block 4: Tinnitus | .015 | .034 | .085 | .689 | .000 | .689 |
| Block 5: Tinnitus X PTSD | -.029 | -.001 | .003 | .760 | .000 | .760 |
| Explicit Memory (*n* = 731) |  |  |  |  |  |  |
| Block 1 |  |  |  |  | .074 | <.001 |
| Age | -.263 | -.016 | .002 | <.001 |  |  |
| Sex | .089 | .165 | .066 | .013 |  |  |
| Block 2: PTSD | **-.266** | **-.005** | **.001** | **<.001** | **.071** | **<.001** |
| Block 3: TBI | .073 | .017 | .008 | .040 | .005 | .040 |
| Block 4: Tinnitus | -.004 | -.004 | .043 | .920 | .000 | .920 |
| Block 5: Tinnitus X PTSD | -.063 | -.001 | .001 | .498 | .001 | .498 |
| Executive Attention (*n* = 731) |  |  |  |  |  |  |
| Block 1 |  |  |  |  | .062 | <.001 |
| Age | .243 | .063 | .009 | <.001 |  |  |
| Sex | -.073 | -.577 | .286 | .044 |  |  |
| Block 2: PTSD | **.242** | **.020** | **.003** | **<.001** | **.058** | **<.001** |
| Block 3: TBI | -.041 | -.041 | .037 | .261 | .002 | .261 |
| Block 4: Tinnitus | .015 | .076 | .185 | .682 | .000 | .682 |
| Block 5: Tinnitus X PTSD | .058 | .004 | .006 | .542 | .000 | .542 |
| Sleep Disturbance (*n =* 697) |  |  |  |  |  |  |
| Block 1 |  |  |  |  | .002 | .478 |
| Age | .046 | .024 | .020 | .225 |  |  |
| Sex | .001 | .009 | .618 | .989 |  |  |
| Block 2: PTSD | **.626** | **.105** | **.005** | **<.001** | **.391** | **<.001** |
| Block 3: TBI | .022 | .045 | .062 | .464 | .000 | .464 |
| Block 4: Tinnitus | .070 | .716 | .318 | .025 | .004 | .025 |
| Block 5: Tinnitus X PTSD | -.017 | -.002 | .011 | .835 | .000 | .835 |
| Pain (*n =* 561) |  |  |  |  |  |  |
| Block 1 |  |  |  |  | .022 | .002 |
| Age | .137 | .130 | .040 | .001 |  |  |
| Sex | .051 | 1.502 | 1.234 | .224 |  |  |
| Block 2: PTSD | **.500** | **.151** | **.011** | **<.001** | **.250** | **<.001** |
| Block 3: TBI | .085 | .301 | .132 | .023 | .007 | .023 |
| Block 4: Tinnitus | **.184** | **3.383** | **.690** | **<.001** | **.030** | **<.001** |
| Block 5: Tinnitus X PTSD | .215 | .054 | .024 | .024 | .006 | .024 |
| Functional impairment (*n* = 694) |  |  |  |  |  |  |
| Block 1 |  |  |  |  | .011 | .022 |
| Age | .105 | .217 | .078 | .006 |  |  |
| Sex | -.007 | -.472 | 2.401 | .844 |  |  |
| Block 2: PTSD | **.657** | **.428** | **.019** | **<.001** | **.431** | **<.001** |
| Block 3: TBI | .012 | .094 | .232 | .687 | .000 | .687 |
| Block 4: Tinnitus | **.145** | **5.765** | **1.172** | **<.001** | **.019** | **<.001** |
| Block 5: Tinnitus X PTSD | .203 | .111 | .041 | .007 | .006 | .007 |

*Note*. Significant effects that passed multiple-testing correction are bolded. PTSD = posttraumatic stress disorder; TBI = traumatic brain injury; SE = standard error; β = standardized coefficient; B = unstandardized coefficient

**Table S7**

*Tinnitus X TBI in Predicting Cross-Sectional Health and Functional Outcomes*

| Outcome | β | B | SE | *p* | *ΔR^2^* | *p* |
| --- | --- | --- | --- | --- | --- | --- |
| Working Memory (*n =* 731) |  |  |  |  |  |  |
| Block 1 |  |  |  |  | .048 | <.001 |
| Age | -.219 | -.026 | .004 | <.001 |  |  |
| Sex | .024 | .088 | .131 | .503 |  |  |
| Block 2: PTSD | **-.226** | **-.008** | **.001** | **<.001** | **.051** | **<.001** |
| Block 3: TBI | .061 | .028 | .017 | .094 | .003 | .094 |
| Block 4: Tinnitus | .015 | .034 | .085 | .689 | .000 | .689 |
| Block 5: Tinnitus X TBI | .030 | .014 | .040 | .727 | .000 | .727 |
| Explicit Memory (*n* = 731) |  |  |  |  |  |  |
| Block 1 |  |  |  |  | .074 | <.001 |
| Age | -.263 | -.016 | .002 | <.001 |  |  |
| Sex | .089 | .165 | .066 | .013 |  |  |
| Block 2: PTSD | **-.266** | **-.005** | **.001** | **<.001** | **.071** | **<.001** |
| Block 3: TBI | .073 | .017 | .008 | .040 | .005 | .040 |
| Block 4: Tinnitus | -.004 | -.004 | .043 | .920 | .000 | .920 |
| Block 5: Tinnitus X TBI | .025 | .006 | .020 | .767 | .000 | .767 |
| Executive Attention (*n* = 731) |  |  |  |  |  |  |
| Block 1 |  |  |  |  | .062 | <.001 |
| Age | .243 | .063 | .009 | <.001 |  |  |
| Sex | -.073 | -.577 | .286 | .044 |  |  |
| Block 2: PTSD | **.242** | **.020** | **.003** | **<.001** | **.058** | **<.001** |
| Block 3: TBI | -.041 | -.041 | .037 | .261 | .002 | .261 |
| Block 4: Tinnitus | .015 | .076 | .185 | .682 | .000 | .682 |
| Block 5: Tinnitus X TBI | .008 | .009 | .088 | .920 | .000 | .920 |
| Sleep Disturbance (*n =* 697) |  |  |  |  |  |  |
| Block 1 |  |  |  |  | .002 | .478 |
| Age | .046 | .024 | .020 | .225 |  |  |
| Sex | .001 | .009 | .618 | .989 |  |  |
| Block 2: PTSD | **.626** | **.105** | **.005** | **<.001** | **.391** | **<.001** |
| Block 3: TBI | .022 | .045 | .062 | .464 | .000 | .464 |
| Block 4: Tinnitus | .070 | .716 | .318 | .025 | .004 | .025 |
| Block 5: Tinnitus X TBI | -.018 | -.037 | .150 | .804 | .000 | .804 |
| Pain (*n =* 561) |  |  |  |  |  |  |
| Block 1 |  |  |  |  | .022 | .002 |
| Age | .137 | .130 | .040 | .001 |  |  |
| Sex | .051 | 1.502 | 1.234 | .224 |  |  |
| Block 2: PTSD | **.500** | **.151** | **.011** | **<.001** | **.250** | **<.001** |
| Block 3: TBI | .085 | .301 | .132 | .023 | .007 | .023 |
| Block 4: Tinnitus | **.184** | **3.383** | **.690** | **<.001** | **.030** | **<.001** |
| Block 5: Tinnitus X TBI | .159 | .586 | .306 | .056 | .005 | .056 |
| Functional impairment (*n* = 694) |  |  |  |  |  |  |
| Block 1 |  |  |  |  | .011 | .022 |
| Age | .105 | .217 | .078 | .006 |  |  |
| Sex | -.007 | -.472 | 2.401 | .844 |  |  |
| Block 2: PTSD | **.657** | **.428** | **.019** | **<.001** | **.431** | **<.001** |
| Block 3: TBI | .012 | .094 | .232 | .687 | .000 | .687 |
| Block 4: Tinnitus | **.145** | **5.765** | **1.172** | **<.001** | **.019** | **<.001** |
| Block 5: Tinnitus X TBI | .065 | .532 | .555 | .339 | .001 | .339 |

*Note*. Significant effects that passed multiple-testing correction are bolded. PTSD = posttraumatic stress disorder; TBI = traumatic brain injury; SE = standard error; β = standardized coefficient; B = unstandardized coefficient

**References**

Newman, C. W., Jacobson, G. P., & Spitzer, J. B. (1996). Development of the Tinnitus Handicap Inventory. *Archives of Otolaryngology--Head & Neck Surgery*, *122*(2), 143–148. https://doi.org/10.1001/archotol.1996.01890140029007
